# Efficacy of Artesunate against *Pseudomonas aeruginosa* Biofilm Mediated by Iron

**DOI:** 10.1155/2019/4810217

**Published:** 2019-11-11

**Authors:** Lei Bao, Jinjin Guo, Lei Feng, Xianjie Zhou, Qi Lu

**Affiliations:** Department of Neonatology, Chongqing Key Laboratory of Pediatrics, Ministry of Education Key Laboratory of Child Development and Disorders, National Clinical Research Center for Child Health and Disorders, China International Science and Technology Cooperation Base of Child development and Critical Disorders, Children's Hospital of Chongqing Medical University, Chongqing 400014, China

## Abstract

*Pseudomonas aeruginosa* is capable of causing a variety of chronic infections due to the formation of biofilms. Iron is essential for growth of *Pseudomonas aeruginosa*, and therapies that interfere with iron may help treat *P. aeruginosa* infections. Herein, we investigated whether artesunate, which is a type of iron-dependent drug, could influence *Pseudomonas aeruginosa* biofilm formation and structure, including the underlying mechanisms. Artesunate could enhance twitching motility significantly and decrease the proportion of surviving cells in *Pseudomonas aeruginosa* biofilms in a dose-dependent manner. Artesunate treatment also reduced biofilm thickness, diffusion in the biomass, and the content of Fe(II). However, changes in biofilm structure and ion concentration were very similar following treatment with 512 *μ*g/ml and 1024 *μ*g/ml artesunate. Interestingly, both biofilm structure and surviving cell fraction were recovered after iron supplementation. These results suggest that artesunate interferes with *Pseudomonas aeruginosa* biofilms by decreasing bacterial viability and enhancing twitching motility in an iron-independent manner.

## 1. Introduction

*Pseudomonas aeruginosa*is an ubiquitous Gram-negative opportunistic pathogen that has the ability to thrive in most natural and man-made environments [1]. It is responsible for chronic lung infections in over 90% of cystic fibrosis (CF) patients [2]. Patients in intensive care units were particularly vulnerable to *Pseudomonas aeruginosa*, which accounts for ∼200,000 nosocomial infections per year worldwide [3]. These infections were considered to be linked to the formation of biofilms, which make it difficult to eradicate by antibiotic intervention since bacterial cells living as biofilms are much more tolerant to antibiotics than their planktonic counterparts. Indeed, the minimal inhibitory concentration (MIC) of antimicrobial agents can be increased 100- to 1000-fold for bacteria reproduction in biofilms, resulting in high morbidity and mortality among infected patients [4]. Thus, there is an urgent need to develop alternative treatment regimens to treat/cure infections and improve disease prognosis.

Iron is an important environmental parameter which played a central role in the development and maintenance of *Pseudomonas aeruginosa* infections; *Pseudomonas aeruginosa* needs iron to sustain growth and virulence [5]. Transcriptome studies have shown that a large number of genes are regulated by iron. The sputum of CF patients contains elevated levels of ferrous iron that correlate with disease severity [6]. Iron limitation compromises biofilm formation, and human lactoferrin inhibits *Pseudomonas aeruginosa* biofilm formation by sequestering iron from siderophores [7]. The combined application of tobramycin and FDA-approved ferric iron chelators could reduce biofilm biomass by ∼90% [8]. There were reasons to be optimistic regarding the efficacy of therapeutics that interfere with Fe(III) acquisition at early stages of infection [9, 10]. Hence, the dependence of bacteria on iron acquisition for biofilm formation has led to its identification as a novel therapeutic to eliminate *Pseudomonas aeruginosa* infections within the host, particularly for CF patients.

Artesunate (AS) is a type of iron-dependent drug activated by cleavage of the endoperoxide bridge in the presence of ferrous iron or haem via a Fenton-type reaction, which generates reactive oxygen species (ROS) and carbon-centred radicals that are highly toxic to the intraerythrocytic parasite [11]. In recent years, it has been proved that artesunate has antibacterial effects on a variety of bacteria and it could increase the sensitivity of methicillin-resistant *Staphylococcus aureus* (MRSA) to antibiotics such as xylopectin [12]. When combined with ceftriaxone sodium, artesunate has certain antibacterial sensitization effect on clinical isolates of *Pseudomonas aeruginosa* [13]. In the treatment of candida albicans biofilm infection, the ratio of the antibiofilm effect of miconazole combined with artesunate and alone was 1.69 [14]. But the exact mechanism for these synergies is still unclear. So far as we are aware, no study on the effect of artesunate on *Pseudomonas aeruginosa* biofilms has been reported. In this study, we hypothesize that some of its antibiofilm properties may be contributed by artesunate binding to ferrous iron and sought to elucidate the antibiofilm activity of artesunate and its underlying mechanisms.

## 2. Materials and Methods

### 2.1. Bacterial Strains and Chemicals

Bacteria were streaked from a −80°C glycerol stock onto a Luria-Bertani agar (LB) (BD Difco, USA) plate and a single colony was inoculated into LB media and incubated at 37°C for 14 h with 200 rpm shaking [15]. Stock solutions of artesunate (TCI, Tokyo, Japan)were prepared in dimethyl sulfoxide (DMSO) (Sigma, St Louis, MO, USA), and 3-(2-pyridyl)-5, 6-diphenyl-1,2,4-triazine-p,p-disulphonic acid monosodium salt hydrate was also supplied by Sigma. The iron standard was ferrous ammonium sulphate (Kelon Chemical, Chengdu, China). Wild-type *Pseudomonas aeruginosa* PAO1 cells were kindly provided by Dr. Zhijun Song (Department of Clinical Microbiology, University of Copenhagen, Copenhagen, Denmark).

### 2.2. Twitch Motility Assays

Cells were stab-inoculated with a toothpick through a thin (∼3 mm) 1% LB agar layer on the bottom of a Petri dish. After incubation for 24−48 h at 30°C, a hazy zone of growth was observed at the interface between the agar and the polystyrene surface. The ability of bacteria to twitch strongly on the polystyrene surface was examined by removing the agar, eliminating unattached cells with a stream of tap water, and staining attached cells with a 1% (w/v) crystal violet solution [16]. Each assay was performed in triplicate and repeated three times.

### 2.3. Growth Assays

PAO1 in the exponential phase of growth were diluted in LB broth to reach a concentration of 1.0 × 10^6^ CFU/mL. Growth curves of PAO1 were cultivated in different concentrations of artesunate (0, 64, 128, 256, 512 and 1024 *μ*g/ml) that were measured at 600 nm at 2 h intervals up to 24 h with a spectrophotometer (UV-1800, Shimadzu, Tokyo, Japan). All experiments were conducted three times independently [17].

### 2.4. Biofilm Formation Protocol

Bacterial strains were cultured at a 1 : 5 dilution of LB broth (20% LB) and grown at neutral pH at 37°C overnight, harvested by centrifugation (3000 ×*g*, 4°C, 10 min), resuspended in sterile saline, and adjusted to a density of 10^9^ colony-forming units (CFU)/ml by measuring the absorbance at 600 nm. A 10 mm diameter membrane was seeded with 1 ml of overnight culture and grown for 3 days to allow biofilms to form. The media was replaced after 48 h, and on the third day, artesunate was put into treatment groups for 12 h incubation [18].

### 2.5. Assessment of *P. aeruginosa* Biofilm-Mediated Resistance

After 3 days in culture, biofilms were tested for drug susceptibility. Membranes were washed twice with phosphate-buffered saline (PBS) to remove planktonic cells. Then, it was treated with different concentrations of artesunate in LB medium, ranging from 64 to 1024 *μ*g/ml; the biofilms were incubated for an additional 12 h at 37°C. Coverslips were then rinsed three times with PBS and subsequently sonicated for 5 min (Tomy UD-201, Tokyo, Japan) and vortexed for 1 min at room temperature. Bacteria were harvested, diluted and plated on LB agar, and incubated the plates overnight, and then, the colonies were counted [19].

### 2.6. Biofilm Ferrozine Assay

The Fe^2+^ concentration was measured by colorimetric assay [20]. Coverslips were then rinsed three times with PBS and subsequently added to 1 ml of 0.5 M HCl and sonicated for 30 min at room temperature. Next, 800 *μ*l of biofilm filtrate was added to 200 *μ*l of ferrozine solution (10 g/l ferrozine in 50 mM of HEPES buffer, pH 7) and incubated for 1 h. The absorbance was measured at 562 nm to quantify Fe^2+^. Ferrous ammonium sulphate was used as the iron standard. Standards (0−100 *μ*M) were also prepared and analysed as above, and a standard curve was used to determine Fe^2+^ concentrations in samples.

### 2.7. Biofilm Staining and Confocal Laser Scanning Microscopy (CLSM)

Biofilms were stained with SYTO 9-propidium iodide live/dead BAC light bacterial stain following the kit instructions (Invitrogen Molecular Probes, USA), and CLSM was performed as describe previously [21]. Biofilms were incubated for 30 min at room temperature in the dark and then washed with PBS. After staining, treated biofilms were observed using a CLSM system (Radiance 2000, Bio-Rad, UK) comprising a microscope (Nikon, Japan) and a krypton-argon mixed-gas laser source. Signals were recorded in the green channel (excitation 488 nm, emission 515/30 nm) and the red channel (excitation 568 nm, emission 600/50 nm).

### 2.8. Quantification with Structural Parameters

Stacks of horizontal-plane images captured by CLSM were subjected to quantitative image analysis using COMSTAT software [22]. We selected five parameters for determination: total biomass, maximum thickness, average thickness, roughness coefficient, and average diffusion distance [22].

### 2.9. Statistical Analysis

Statistical analyses were performed using SPSS 22.0 (IBM, Inc, NY, USA). All data were expressed as the mean ± SD and statistically analysed by independent sample one-way ANOVA. Differences were considered to be statistically significant when *p* < 0.05.

## 3. Results

### 3.1. Impact of Artesunate on Twitch Motility

**Figure 1:**
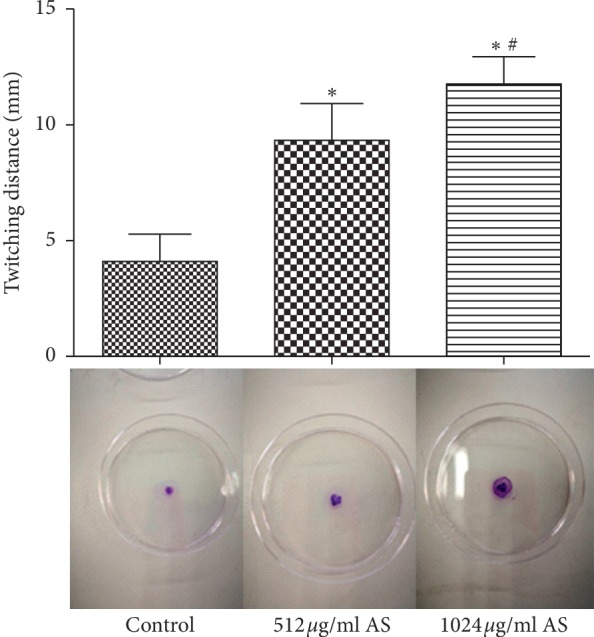
The diameter formed by the twitching motility of *Pseudomonas aeruginosa* in presence of different concentrations of artesunate (AS). ^*∗*^*p* < 0.05 versus the control; ^#^*p* < 0.05 versus the 512 *μ*g/ml AS.

Twitch motility is an important step in the formation of microscopic bacterial colonies and biofilms. We observed that the distance of twitch movement in the artesunate treatment groups was greater than in the control group and the distance in the 1024 *μ*g/ml group was greater than that in the 512 *μ*g/ml group (Figure 1).

### 3.2. Effects of Artesunate on Planktonic Cell Growth

**Figure 2:**
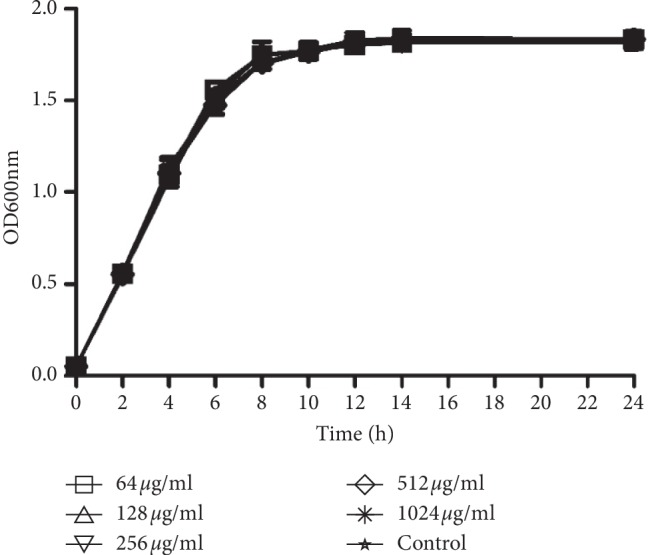
Effects of artesunate on planktonic growth of PAO1. Growth of *Pseudomonas aeruginosa* at different concentrations of artesunate for 24 h incubation.

Since artesunate is reported to possess antiviral and antifungal activities, we assessed the antimicrobial activity of artesunate against PAO1 using growth curves. However, growth curves for a series of artesunate concentrations were not significantly different in lag, exponential, or stationary phases during 24 h of incubation. These results suggest that the growth rate of planktonic PAO1 cells was not influenced by the addition of artesunate up to 1024 *μ*g/ml (Figure 2).

### 3.3. Antibacterial Susceptibility Assay of Artesunate on *P. aeruginosa* Biofilm

**Figure 3:**
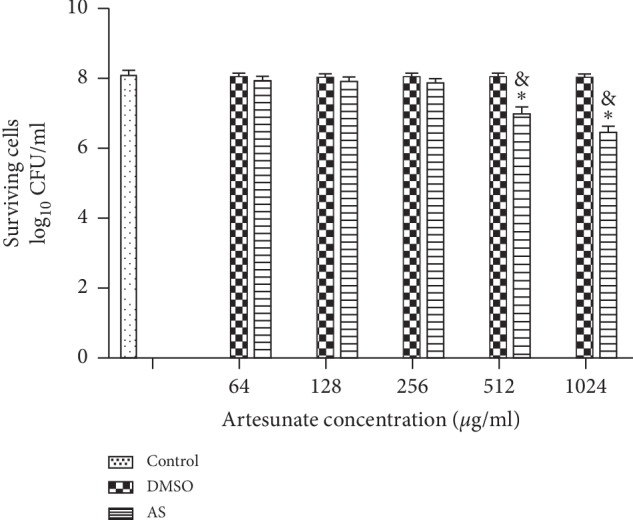
Killing of PAO1 cells by artesunate. Cells from biofilm cultures were treated with artesunate (AS) for 12 h and then plated for colony counting. ^*∗*^*p* < 0.05 versus the control; ^＆^*p* < 0.05  versus the DMSO.

We investigated the effects of different concentrations of artesunate on mature PAO1 biofilms and found that surviving cells in the 512 *μ*g/ml and 1024 *μ*g/ml artesunate treatment groups, which served as experimental groups in further research, were significantly less abundant than in the control group (*p* < 0.05). Surviving cells in other treatment groups were only slightly fewer than in the control group, and the difference was not significant (*p* > 0.05; Figure 3).

### 3.4. Ferrous Production in Biofilm

**Figure 4:**
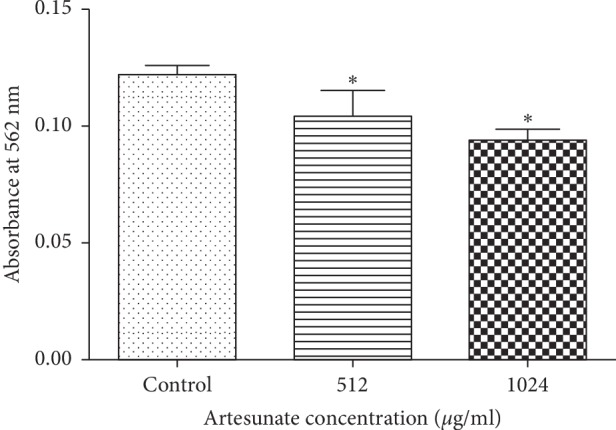
Ferrous iron in biofilms measured by ferrozine assay ferrozine-Fe^2+^ complex quantified in the presence of artesunate. The amount of ferrozine-Fe^2+^ complex was determined at OD 562 nm. ^*∗*^*p* < 0.05 versus the control.

It is well known that artesunate is a type of Fe^2+^-dependent drug. Thus, to confirm the direct effect of artesunate on ferrous iron in biofilms, ferrozine assays were performed to test Fe^2+^ concentrations in different groups. Compared with the control group, Fe^2+^ concentrations in both 512 *μ*g/ml and 1024 *μ*g/ml treatment groups were decreased (*p* < 0.05), but the difference was not significant between the two artesunate groups (*p* > 0.05) (Figure 4).

### 3.5. Effects of Combined Treatment with Artesunate and Iron

**Figure 5:**
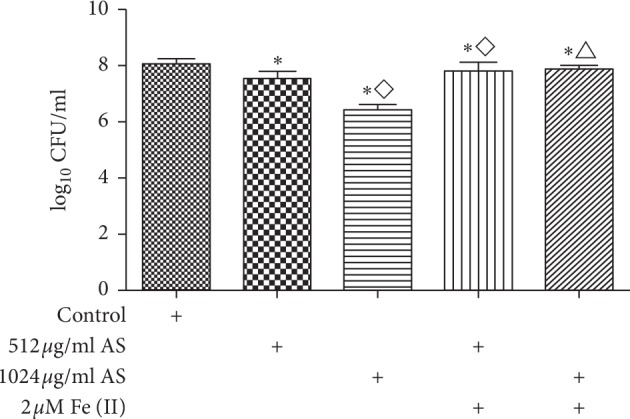
The number of viable cells in *Pseudomonas aeruginosa* biofilm in presence of different concentrations of artesunate (AS) combine ferrous iron ^*∗*^*p* < 0.05 versus the control; ⋄*p* < 0.05 versus 512 *μ*g/ml AS, △ versus 1024 *μ*g/ml AS.

The effects of combined treatment with artesunate and iron on PAO1 cells were investigated, and the results showed that the number of live bacteria in biofilms was higher in the combined treatment group than in the equivalent artesunate-only group (*p* < 0.05), but lower than in the control group (*p* < 0.05) (Figure 5).

### 3.6. Biofilm Structure

**Figure 6:**
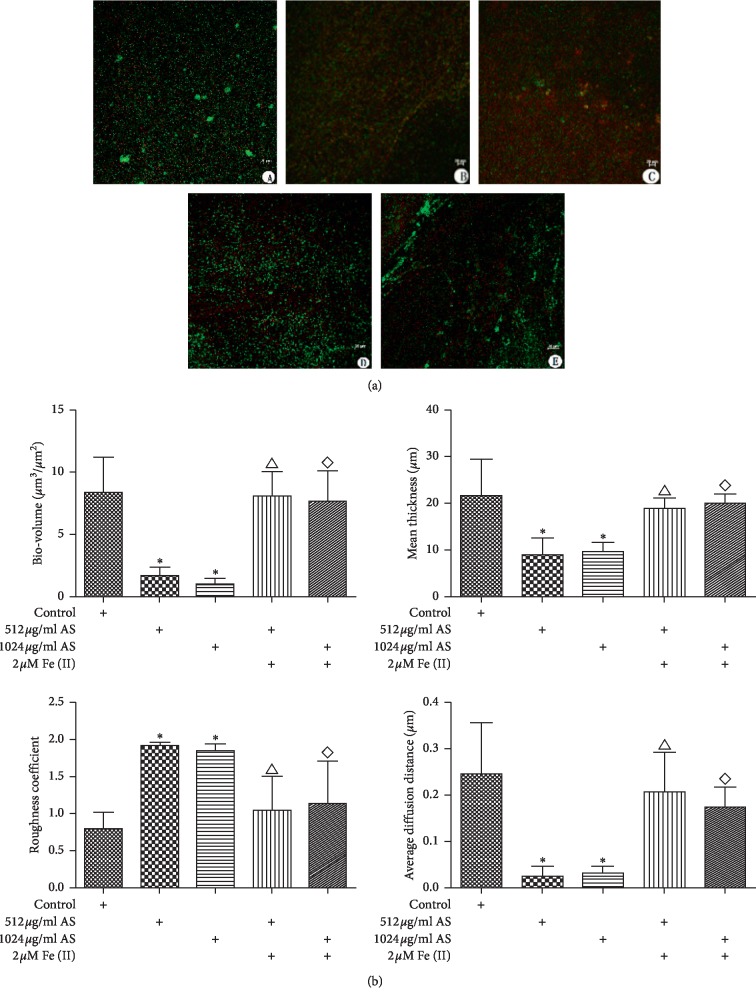
(a) Confocal laser scanning images. Three days later, the biofilm was exposed to different concentrations of artesunate (AS) combine ferrous iron for 12 h (A) Control, (B) 512 *μ*g/ml AS, (C) 1024 *μ*g/ml AS, (D) 512 *μ*g/ml AS + Fe, (E) 1024 *μ*g/ml AS + Fe(II). (b) COMSTAT analysis of biofilms parameters. Three days later, the biofilm was exposed to different concentrations of artesunate (AS) combined with ferrous iron for 12 h. ^*∗*^*p* < 0.05 versus the control; △*p* < 0.05 versus 512 *μ*g/ml AS; ⋄versus 1024 *μ*g/ml AS.

After treatment with artesunate, biofilms appeared more diffuse, and the number of attached bacteria diminished. Furthermore, since SYTO 9 penetrates all bacterial membranes and stains cells green, whereas propidium iodide only penetrates cells with damaged membranes and viable and nonviable cells could be distinguished. Following artesunate treatment, the number of viable (green) cells was decreased relative to nonviable cells (red) [23]. Furthermore, a combination of artesunate and iron resulted in dense bacterial growth and growth in the number of live bacteria (Figure 6(a)). For further investigation of the effect of artesunate on PAO1 biofilm architecture, COMSTAT image analysis was employed to evaluate biofilm parameters. The results of CLSM revealed that the number of viable bacteria in the control group was greater than that in the 512 *μ*g/ml and 1024 *μ*g/ml artesunate treatment groups. Furthermore, the total biofilm biomass, average and maximum biomass thickness, and average diffusion distance were decreased in the treatment groups compared to the control group, but the roughness coefficient was greater. The combined artesunate and iron treatment increased the total biofilm biomass, the average and maximum biofilm thickness, and the average diffusion distance, as well as the roughness coefficient, although the difference was not significant (*p* > 0.05; Figure 6(b)).

## 4. Discussion

Biofilm-associated infections are often difficult to treat due to multiple drug resistance; hence, it is important to identify new and effective molecules targeting bacterial biofilm formation and cell viability. Iron is proved essential for biofilm growth, especially at levels in excess, is required for growth, and promotes biofilm formation by signaling a transition from motile to sessile [8, 24]. We found iron ions in biofilms mainly existed in the form of reduced divalent iron [25]. In the present study, the relationship between the antibacterial properties of artesunate and PAO1 biofilms is investigated in the context of bacterial clearance rate, twitch motility, ferrous iron content, and biofilm structure, since these mechanisms are currently poorly understood. Defining these mechanisms could provide a better understanding of bacterial responses to artesunate, thereby facilitating the development of artesunate-based formulations suitable as effective biofilm inhibitors.

In this study, we provide the first-ever evidence for artesunate-decreased PAO1 biofilm formation at a concentration of 512 and 1024 *μ*g/ml in a dose-dependent manner. However, artesunate had no effect on planktonic *Pseudomonas aeruginosa* cells, consistent with previous findings [13], indicating that artesunate does not destroy bacteria directly. It is reported that chelating divalent cationic iron facilitates killing of *Pseudomonas aeruginosa* biofilm cells [26]. Artesunate reacts with divalent iron ions to produce ROS and free radicals that damage bacteria. In the present study, ferrous iron decreases biofilms at 3 days after artesunate treatment, but when iron ions are added to biofilms, the surviving cell fraction is increased. This suggests that artesunate can kill bacteria via an iron-dependent mechanism. Another study showed that artemisinin and its derivatives with a similar oxygen bridge structure can compete for ferrous ions with drug-resistant *Mycobacterium tuberculosis* strains and thereby induce bacterial lysis [13].

Adhesion is considered to be the first step in the development of bacterial biofilms and a critical step in initiating infection [27]. Twitching motility is important for migration of cells along surfaces to form multicellular aggregates. The active expansion of *Pseudomonas aeruginosa* biofilms is a complex, multicellular, collective behaviour under the mediation of twitching motility [28, 29]. Excessive twitching movement would inhibit the formation of biofilms [30]. We therefore investigated whether artesunate affected the twitching motility of *Pseudomonas aeruginosa* cells. The results showed the artesunate group has the larger twitch movement diameter than the control group in a concentration-dependent manner, suggesting that artesunate can promote type IV pili motility and inhibit the formation of macrocolonies, thereby preventing differentiation into a three-dimensionally biofilm development involves specific stages such as initiation (including adhesion), maturation, and detachment [31]. Because the maturation period of a *Pseudomonas aeruginosa* biofilm is approximately 3−5 days [32], we examined biofilms of *Pseudomonas aeruginosa* 3 days after treatment. The results suggest that artesunate disrupted biofilm structure and formation. After artesunate treatment, the roughness coefficient is increased while the average diffusion distance is decreased, indicating that biofilms are sparser with larger pore channels and interstices, allowing easier antimicrobial penetration. Volume measurements indicate that the biofilm structure is simpler and more likely to be affected by the surrounding environment. We concluded that artesunate inhibited biofilm formation of *Pseudomonas aeruginosa* during both the initiation and maturation stages. When iron ions are added, PAO1 biofilms become thicker, with increased ADD and biofilm biomass combined with a reduced roughness coefficient. These results suggest that artesunate disrupts biofilm structure via iron. Consistently, when iron availability is limited, rhamnolipid production increased, resulting in a decrease in average biofilm thickness and an increase in the roughness coefficient [33]. In addition, more Psl polysaccharides are produced under the signal of high iron concentration [34]. Artesunate can also form carbon-centred radicals and reactive oxygen species (ROS). ROS cause disruption to *Pseudomonas aeruginosa* biofilm, and it breaks the biofilm structure by attacking EPS [35].

Artesunate treatment disrupts biofilm formation and decreases the number of viable cells in a dose-dependent manner, but the destruction of biological membranes and the Fe(II) content in groups treated with 512 *μ*g/ml or 1024 *μ*g/ml artesunate are comparable. The reason why bacterial cell killing increased might be that artesunate regulates the distribution of calcium ions both inside and outside the cell, reduces iron ions, and promotes releasing ROS [36].

## 5. Conclusions

These results suggest that artesunate interferes with *Pseudomonas aeruginosa* biofilm formation in a dose-dependent manner by decreasing bacterial cell viability and enhancing twitching motility independently of iron. Therefore, AS could be considered as a candidate for the treatment of *Pseudomonas aeruginosa* biofilm-related infections.

## Data Availability

All data used to support the findings of this study are included within the article.
